# Supplementary material for: Psychosocial impact of the COVID-19 pandemic on 4378 UK healthcare workers and ancillary staff: initial baseline data from a cohort study collected during the first wave of the pandemic
Source: Occup Environ Med. 2021 Jun 27:oemed-2020-107276. doi: 10.1136/oemed-2020-107276 (PMC8245285; doi:10.1136/oemed-2020-107276)
Supplement: Supplementary file 1 [file oemed-2020-107276supp001.pdf]

Supplementary tables

Supplementary table 1 Weighted and unweighted socio-demographics (n=4,378)

Variable		n	Unweighted %	Weighted %
Age (years)				
	≤30	985	23.8	24.2
	31-40	1,138	27.5	30.7
	41-50	979	23.7	23.5
	51-60	861	20.8	20.8
	≥61	171	4.1	4.1
Sex				
	Female	3,485	80.3	74.8
	Male	833	19.2	24.6
	Other	6	0.1	0.2
	Prefer not to say	18	0.4	0.5
Relationship status				
	Married/Civil partnership	1,827	42.3	42.2
	Co-habiting/in a relationship	1,129	26.1	23.2
	Divorced/separated/widowed	251	5.8	6.3
	Single	1,114	25.8	28.3
Ethnicity				
	White	3,263	74.5	53.5
	Black/African/Caribbean/Black British	373	8.5	20.8
	Asian/Asian British	482	11.0	17.4
	Mixed/Multiple racial and ethnic minority groups	173	4.0	3.8
	Other racial and ethnic minority groups[a]	90	2.1	4.5
Country of birth				
	UK	2,974	68.9	61.0
	EU not UK)	525	12.2	9.8
	Other	815	18.9	29.2
Length of time living in the UK				
	<1-2 years	106	8.0	7.8
	3-5 years	163	12.2	10.9
	6-10 years	210	15.8	14.5
	11-20 years	404	30.3	32.5
	21-29 years	223	16.7	17.1
	>30 years	220	16.5	16.5
	Prefer not to say	7	0.5	0.7
Main role				
	Doctor	557	12.9	12.8
	Nurse	1,108	25.6	26.7
	Other clinical	1,306	30.2	28.3
	Non-clinical	1,358	31.4	32.3

a ‘Other racial and ethnic minority groups’ includes the options ‘Arab’ and ‘Any other ethnic background’.

Supplementary table 2 Socio-demographics of short-survey-only and short-and-long-survey participants

Variable		Short-survey only n (%)[a]	Short- and long-survey n (%)[a]	Significant differences p=
Age (years)				
	≤30	464 (23.4)	521 (25.1)	0.31
	31-40	574 (31.0)	564 (30.4)	
	41-50	522 (24.9)	457 (22.0)	
	51-60	419 (16.7)	442 (18.5)	
	≥61	86 (4.0)	85 (4.0)	
Sex				
	Female	1,691 (72.1)	1,791 (74.8)	0.001
	Male	473 (27.5)	360 (24.6)	
	Other	1 (<1)	5 (<1)	
	Prefer not to say	10 (<1)	8 (<1)	
Relationship status				
	Married/Civil partnership	930 (42.3)	897 (42.1)	0.24
	Co-habiting/in a relationship	538 (21.9)	591 (24.7)	
	Divorced/separated/widowed	128 (6.8)	123 (5.9)	
	Single	566 (29.1)	546 (27.4)	
Ethnicity				
	White	1,530 (48.4)	1,685 (59.4)	<0.0001
	Black/African/Caribbean/Black British	219 (22.7)	154 (19.0)	
	Asian/Asian British	283 (20.0)	194 (14.6)	
	Mixed/Multiple racial and ethnic minority groups	83 (3.5)	90 (4.3)	
	Other racial and ethnic minority groups[b]	55 (5.3)	35 (3.7)	
Country of birth				
	UK	1,440 (58.2)	1,534 (64.2)	0.0007
	EU (not UK)	267 (9.6)	258 (10.0)	
	Other	454 (32.2)	358 (25.8)	
Length of time living in the UK				
	<1-2 years	59 (8.1)	47 (7.4)	0.49
	3-5 years	85 (10.9)	78 (11.0)	
	6-10 years	102 (12.9)	108 (16.7)	
	11-20 years	230 (33.9)	174 (30.7)	
	21-29 years	120 (17.1)	103 (17.2)	
	>30 years	117 (16.2)	103 (16.8)	
	Prefer not to say	5 (1.0)	2 (<1)	
Main role				
	Doctor	316 (13.9)	241 (11.7)	0.001
	Nurse	479 (23.7)	628 (29.9)	
	Other clinical	691 (29.7)	615 (26.8)	
	Non-clinical	677 (32.8)	679 (32.3)	

a Numbers are unweighted, proportions are weighted.

b 'Other racial and ethnic minority groups' includes the options 'Arab' and 'Any other ethnic background'.

Supplementary table 3 Prevalence of mental health outcomes by socio-demographic factors [a]

Characteristic		Probable common mental disorders % (95%CI)	Probable anxiety % (95%CI)	Probable depression % (95%CI)	Probable alcohol misuse % (95%CI)	Probable PTSD % (95%CI)	Perceived moral injury Mean (95%CI)
Age years)		**n=3,785*****	**n=2,369*****	**n=2,368*****	n=2,219	**n=2,351*****	**n=2,304 ****
	≤30	67.5 [63.8, 70.9]	31.3 (27.0, 35.9)	35.3 (30.9, 40.1)	11.7 (9.1, 15.0)	35.8 (31.4, 40.5)	16.0 (15.2, 16.8)
	31-40	60.8 [57.2, 64.2]	24.2 (20.6, 28.1)	30.6 (26.6, 34.8)	11.0 (8.7, 13.9)	32.3 (28.3, 36.5)	15.9 (15.0, 16.7)
	41-50	58.5 [54.4, 62.4]	22.0 (18.0, 26.5)	23.6 (19.6, 28.2)	9.2 (7.0, 12.1)	30.6 (25.9, 35.7)	15.6 (14.7, 16.5)
	51-60	49.9 [45.6, 54.2]	17.7 (13.9, 22.3)	20.7 (16.7, 25.3)	10.0 (7.3, 13.5)	21.9 (17.8, 26.6)	14.5 (13.7, 15.2)
	≥61	43.0 [33.1, 53.5]	6.8 (3.4, 13.1)	12.7 (7.4, 20.9)	8.9 (4.4, 17.2)	19.3 (11.2, 31.3)	14.4 (12.5, 16.2)
Sex		**n=3,965*****	**n=2,471***	n=2,466	=2,308	**n=2,447****	n=2,399
	Female	62.2 (60.2, 64.3)	24.8 (22.6, 27.1)	28.8 (26.5, 31.2)	9.5 (8.2, 11.0)	32.4 (30.0, 35.0)	15.3 (14.9, 15.7)
	Male	48.7 (44.5, 52.9)	18.2 (14.4, 22.7)	22.5 (18.4, 27.3)	13.8 (10.7, 17.6)	23.6 (19.2, 28.5)	16.3 (15.3, 17.4)
	Other	58.6 (19.3, 89.3)	32.3 (7.0, 75.0)	32.3 (7.1, 75.0)	0	15.9 (2.0, 63.5)	19.2 (9.7, 28.8)
	Prefer not to say	67.0 (34.7, 88.6)	23.9 (4.9, 65.7)	30.2 (7.5, 70.0)	0	9.8 (1.2, 50.4)	14.1 (9.1, 19.1)
Relationship status		n=3,953	**n=2,463***	**n=2,459*****	**n=2,301*****	**n=2,440*****	**n=2,392****
	Married/Civil partnership	55.7 (52.8, 58.5)	20.0 (17.2, 23.0)	21.9 (19.1, 25.0)	8.6 (7.0, 10.6)	25.1 (22.1, 28.4)	14.9 (14.3, 15.5)
	Co-habiting/in a relationship	61.3 (57.7, 64.8)	26.5 (22.9, 30.5)	30.4 (26.5, 34.5)	14.2 (11.5, 17.6)	34.8 (30.7, 39.2)	15.6 (14.8, 16.3)
	Divorced/separated/widowed	59.8 (51.5, 67.6)	21.4 (14.6, 30.1)	23.3 (16.4, 32.0)	3.6 (1.6, 7.7)	39.7 (30.1, 50.1)	16.3 (14.8, 17.8)
	Single	61.9 (58.2, 65.4)	25.6 (21.6, 30.1)	33.7 (29.3, 38.4)	11.4 (8.8, 14.5)	32.1 (27.8, 36.6)	16.4 (15.5, 17.3)
Ethnicity		n=3,958	n=2,466	n=2,461	**n=2,303*****	n=2,442	**n=2,394*****
	White	59.6 (57.6, 61.4)	22.6 (20.7, 24.7)	27.5 (25.4, 29.7)	14.5 (12.8, 16.4)	29.5 (27.3, 31.7)	14.9 (14.5, 15.4)
	Black/African/ Caribbean/Black British	58.4 (52.8, 63.9)	24.7 (18.5, 32.1)	21.9 (16.0, 29.1)	4.1 (1.9, 8.4)	31.1 (24.3, 38.97)	15.3 (14.1, 16.4)
	Asian/Asian British	55.9 (50.8, 60.8)	24.2 (18.9, 30.5)	30.6 (24.6, 37.2)	4.9 (2.5, 9.3)	32.1 (26.1, 38.8)	18.0 (16.5, 19.5)
	Mixed/Multiple ethnic groups	65.8 (57.7, 73.0)	26.4 (18.3, 36.5)	31.3 (22.4, 41.7)	8.7 (4.3, 16.9)	33.2 (24.2, 43.5)	15.5 (13.8, 17.1)
	Other racial and ethnic minority groups	59.6 (46.7, 71.3)	17.0 (8.4, 31.5)	33.3 (18.9, 51.7)	0	25.8 (13.4, 43.9)	16.3 (13.4, 19.2)
Country of birth		n=3,943	=2,456	n=2,451	**n=2,293*****	n=2, 432	n=2,385
	UK	58.7 (56.5, 60.9)	22.7 (20.5, 25.0)	26.0 (23.7, 28.4)	13.0 (11.4, 14.9)	29.1 (26.7, 31.7)	15.2 (14.8, 15.7)
	EU not UK)	63.9 (59.1, 68.4)	23.6 (18.8, 29.3)	29.7 (24.4, 35.7)	7.1 (4.4, 11.4)	33.5 (27.7, 39.8)	16.2 (15.0, 17.3)

	Other	58.0 (53.7, 62.2)	24.5 (19.8, 29.8)	29.5 (24.6, 35.0)	4.8 (3.1, 7.3)	32.0 (28.1, 32.5)	16.1 (15.1, 17.2)
**Length of time living in the UK**		n=1,176	n=674	n=672	n=627	n=666	**n=646****
	<1-2 years	69.4 (58.4, 78.6)	29.1 (17.4, 44.4)	40.3 (27.0, 55.2)	7.0 (2.9, 16.3)	34.8 (22.5, 49.6)	18.4 (15.3, 21.5)
	3-5 years	68.7 (59.8, 76.5)	27.1 (18.6, 37.6)	28.6 (19.8, 39.3)	4.4 (1.8, 10.5)	26.8 (18.4, 37.4)	17.8 (15.2, 20.3)
	6-10 years	57.6 (49.4, 65.4)	25.6 (17.8, 35.4)	29.7 (21.5, 39.5)	8.3 (4.3, 15.3)	29.7 (21.4, 39.7)	16.0 (13.7, 18.3)
	11-20 years	60.5 (54.3, 66.5)	24.2 (17.5, 32.6)	27.1 (20.4, 35.0)	5.3 (2.8, 9.9)	37.7 (29.8, 46.2)	16.0 (14.4, 17.5)
	21-29 years	55.1 (46.6, 63.3)	20.2 (13.0, 30.1)	32.8 (23.0, 44.4)	5.7 (2.5, 12.5)	26.2 (17.8, 36.9)	15.6 (13.9, 17.2)
	>30 years	50.8 (42.4, 59.2)	19.1 (11.6, 29.9)	23.2 (14.5, 35.0)	2.4 (0.8, 7.3)	30.0 (20.3, 42.0)	14.5 (13.1, 15.9)
	Prefer not to say	61.5 (20.0, 91.1)	35.0 (4.6, 85.6)	35.1 (4.7, 85.7)	7.0 (2.9, 16.3)	70.7 (17.9, 96.4)	14.6 (10.0, 19.1)
**Main role**		**n=3,964 *****	**n=2,480 *****	**n=2,465*****	n=2,307	**n=2,446*****	**n=2,398****
	Doctor	46.9 (41.8,52.0)	13.6 (9.7, 18.9)	15.4 (11.4, 20.4)	10.2 (6.9, 14.8)	18.3 (13.8, 23.8)	14.4 (13.4, 15.4)
	Nurse	68.1 (64.6,71.3)	30.2 (26.3, 34.5)	33.1 (29.0, 37.4)	10.2 (8.0, 12.8)	36.5 (32.3, 40.9)	17.4 (16.5, 18.3)
	Other clinical	59.5 (56.1,62.9)	19.5 (16.3, 23.2)	24.0 (20.5, 28.2)	8.2 (6.3, 10.7)	29.0 (25.2, 33.3)	15.2 (14.4, 16.0)
	Non-clinical	55.6 (52.2,59.1)	23.8 (20.4, 27.5)	29.3 (25.6, 33.2)	12.6 (10.2, 15.5)	30.0 (28.1, 32.5)	14.6 (14.0, 15.3)

a Numbers are unweighted, proportions are weighted.

Differences within categories are statistically significant at: *p<0.05, **p<0.01, ***p<0.001
